# Ribosome Profiling Reveals Genome-wide Cellular Translational Regulation upon Heat Stress in *Escherichia coli*

**DOI:** 10.1016/j.gpb.2017.04.005

**Published:** 2017-10-12

**Authors:** Yanqing Zhang, Zhengtao Xiao, Qin Zou, Jianhuo Fang, Qifan Wang, Xuerui Yang, Ning Gao

**Affiliations:** 1 MOE Key Laboratory of Protein Sciences, Beijing Advanced Innovation Center for Structural Biology, Tsinghua-Peking Center for Life Sciences, School of Life Sciences, Tsinghua University, Beijing 100084, China; 2 MOE Key Laboratory of Bioinformatics, Tsinghua-Peking Joint Center for Life Sciences, Center for Synthetic and Systems Biology, School of Life Sciences, Tsinghua University, Beijing 100084, China; 3 State Key Laboratory of Membrane Biology, Peking-Tsinghua Center for Life Sciences, School of Life Sciences, Peking University, Beijing 100871, China

**Keywords:** Ribosome profiling, Translation regulation, RNA-Seq, Heat shock response, Transcription regulation

## Abstract

**Heat shock response** is a classical stress-induced regulatory system in bacteria, characterized by extensive transcriptional reprogramming. To compare the impact of heat stress on the transcriptome and translatome in *Escherichia coli*, we conducted **ribosome profiling** in parallel with **RNA-Seq** to investigate the alterations in transcription and translation efficiency when *E. coli* cells were exposed to a mild heat stress (from 30 °C to 45 °C). While general changes in ribosome footprints correlate with the changes of mRNA transcripts upon heat stress, a number of genes show differential changes at the transcription and translation levels. Translation efficiency of a few genes that are related to environment stimulus response is up-regulated, and in contrast, some genes functioning in mRNA translation and amino acid biosynthesis are down-regulated at the translation level in response to heat stress. Moreover, our ribosome occupancy data suggest that in general ribosomes accumulate remarkably in the starting regions of ORFs upon heat stress. This study provides additional insights into bacterial gene expression in response to heat stress, and suggests the presence of stress-induced but yet-to-be characterized cellular regulatory mechanisms of gene expression at translation level.

## Introduction

Heat shock response is one of the most characterized stress response pathways. Upon temperature upshift, cells respond by sharply elevating transcription of many genes involved in thermo-protection and protein homeostasis, a majority of which encode various heat-shock proteins (HSPs) [1], [2], [3], [4]. The transcriptional regulation of heat shock response has been well studied using microarray or RNA-Seq based approaches [5], [6]. At molecular level, heat shock response is initiated by binding of heat-specific transcription factors to the heat-shock elements or promoters of HSP genes. In *Escherichia coli*, an alternative sigma factor σ^32^ is the major player of transcriptional control of HSP genes [7]. The most deleterious effect of heat shock is unfolding and aggregation of proteins. As a result, unfolded proteins serve as a signal to trigger the onset of heat shock response and regulate the response through a chaperone titration mechanism.

Under normal condition, proteins of σ^32^ are sequestered by chaperone systems (DnaK–DnaJ in *E. coli*), which keep them in an inactive form or target them to degradation; upon temperature upshift, accumulated unfolded proteins compete for chaperones and therefore release σ^32^ into active form [8]. As a countermeasure, molecular chaperones and proteolytic machines, which constitute a significant portion of the HSPs, are dramatically up-regulated to remove the intermediate threats from unfolded and aggregated proteins [8]. Once unloaded chaperones begin to accumulate, either from excessive synthesis or alleviated proteotoxicity, factors of σ^32^ are again inactivated by chaperones [8]. In this means, the balance between chaperones and unfolded proteins elegantly control the onset and duration of the heat shock response.

However, the regulation of gene expression at translational level during heat shock response is not entirely clear yet. In recent years, ribosome profiling has been widely used for the genome-wide analysis of translational regulation in many organisms, including *E. coli*, yeast, and mammals [9], [10], [11]. To perform ribosome profiling, ribosome-protected mRNA fragments (RPFs) are purified after RNase digestion and rRNA removal. The cDNA library prepared from the resulting RPFs is subjected to deep sequencing, in parallel with the RNA-Seq for profiling the total mRNAs from the same biochemical preparation. Ribosome profiling is a powerful tool that can be used to evaluate translation efficiency (TE) and identify previously-unknown translational events [12].

In the current study, by taking advantage of ribosome profiling, we investigate the ribosome occupancy along mRNAs to evaluate the translational regulation in *E. coli* upon heat stress. We find that in general the changes of ribosome footprints almost track with the changes of mRNA transcripts upon heat stress. But interestingly, a number of genes display up- or down-regulated TE, while their transcription levels remain stable or even change in an opposite direction. Moreover, we also observe that a predominant effect of heat stress on translation is the accumulation of ribosomes in the initiation regions of ORFs. This study provides preliminary insights and clues for the future study of stress-induced translation control in bacteria.

## Results and discussions

### Simultaneous examination of changes in transcriptome and translatome upon heat stress

**Supplementary Figure S1:**
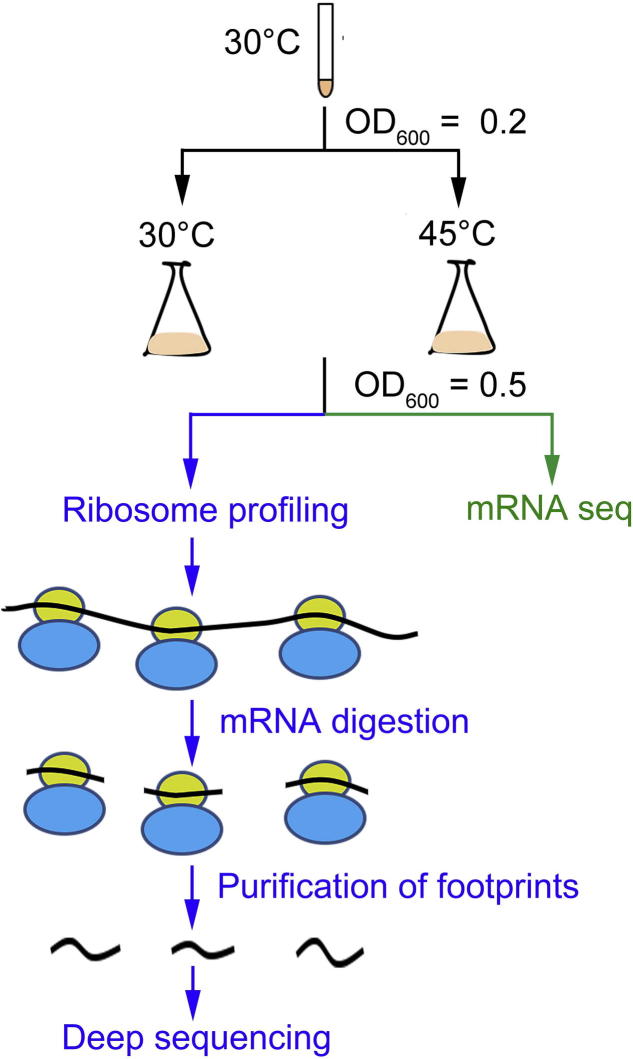
**Flow chart of experimental design**A flow chart of experimental design from cell culture to deep sequencing (heat shock treatment, total mRNA-Seq and ribosome profiling) is illustrated.

To examine the effect of heat stress on transcription and translation, the early log-phase *E. coli* cells pre-cultured at 30 °C were further cultured at 45 °C (a non-lethal heat stress). Two sets of parallel experiments were conducted to analyze the transcriptome and translatome in the same two populations of *E. coli* cells (30 °C *vs.* 45 °C) (Figure S1). One set was used for deep sequencing of cellular total mRNAs to monitor the transcription regulation, and the other set was used for ribosome profiling to quantify RPFs, aiming at exploring the translation regulation upon heat shock. The RNA-Seq transcripts or ribosome footprints produced a large number of reads, ranging from 36,308,125 to 43,500,137 per cDNA library (Table S1) for the four datasets. After filtering, 2,401,035 and 1,663,447 reads were obtained for RPF samples at 30 °C and 45 °C, respectively, while 32,917,372 and 39,493,934 mRNA-seq reads from samples at 30 °C and 45 °C, respectively, were kept for subsequent analysis. These reads were mapped to the reference transcriptome data of *E. coli*. As a result, we obtained 3688 and 3782 mapped genes for RPF samples at 30 °C and 45 °C, respectively. Similarly, 4276 and 4285 mapped genes were obtained for mRNA samples at 30 °C and 45 °C, respectively.

### Ribosome-protected mRNA footprints generally trail transcript abundance

**Figure 1:**
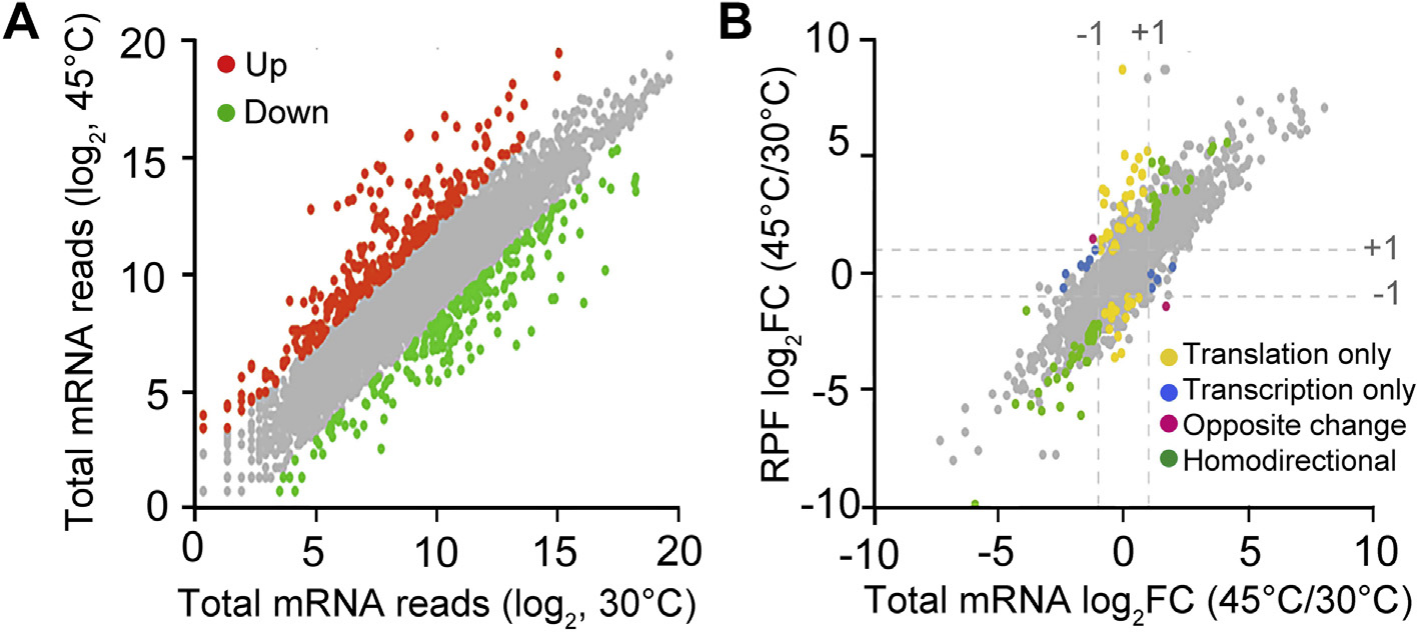
**Simultaneous monitoring of the changes in transcriptome and translatome in *E. coli* upon heat stress** **A.** Comparison of the total mRNA transcripts between 45 °C and 30 °C conditions. Red dots represent the genes with log_2_FC (45 °C/30 °C) >2; green dots represent the genes with log_2_FC < −2. **B.** Plot of the FC (log_2_FC) of RPFs (45 °C/30 °C) against FC of respective mRNAs (45 °C/30 °C). The four dash lines indicate log_2_FC values −1 or +1. FC, fold change; RPF, ribosome-protected fragment.

**Supplementary Figure S2:**
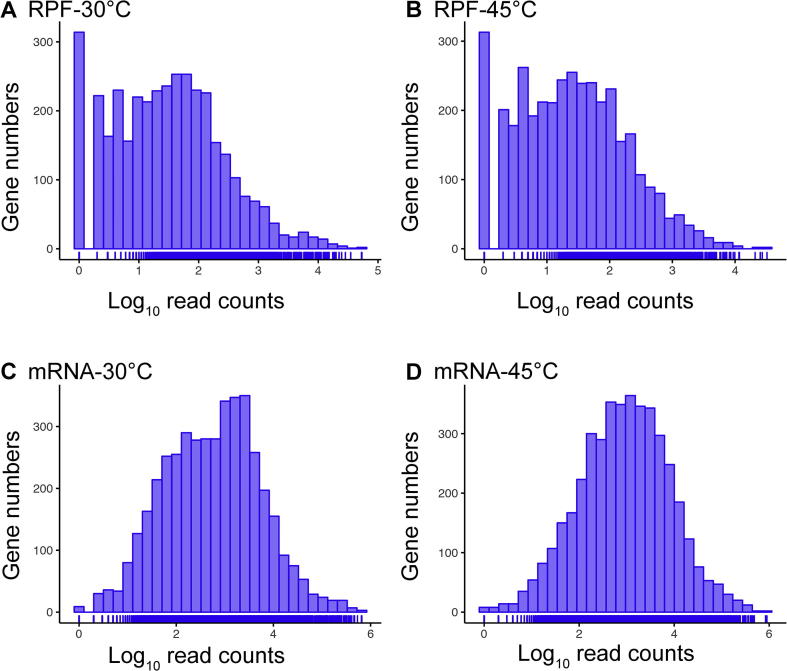
**Distribution of genes in terms of read counts**The distribution of genes in terms of read counts in the four samples was shown for RPF-30 °C (**A**), RPF-45 °C (**B**), mRNA-30 °C (**C**), and mRNA-45 °C (**D**), respectively.

To explore how *E. coli* deals with heat stress, we examined the relative variations at both transcription and translation levels between normal and heat-treated conditions. The Xtail package [13] was used to normalize the read counts for mapped genes and to identify differential translation events. The distribution of genes in terms of read counts is shown in Figure S2. We focused our analysis on 2939 genes with mean read counts >5 (Table S2). As expected, upon temperature upshift, an extensive transcriptional reprogramming of genes in *E. coli* cells was observed, with selected sets of genes falling into the categories of up- and down-regulated groups (Figure 1A). Using Xtail, these genes were classified into distinct groups according to the changes in expression levels (groups were separated with log_2_ fold change of −1 or +1) between normal and heat stress conditions (Figure 1B). For most genes, the fold changes in the mRNA and RPF levels could be well-aligned (gray dots), or closely aligned (green dots), indicating that for these genes the transcription regulation is the major component of heat-shock response. In contrast, a number of genes appear to be dominated by translational (yellow dots) regulation only. Interestingly, two genes (Left, *yfeA*; Right, *pyrL*) show opposite changes at transcriptional and translational levels (red dots).

The detailed quantifications of fold changes for all genes in terms of mRNA expression and TEs are provided in Table S2. With the adjusted *P* < 0.1, genes with differential translational regulation are listed in Table S3, including 58 genes with up-regulated TE and 57 genes with down-regulated TE. In summary, the general changes in the ribosome occupancy upon heat treatment trail the transcript production for most of genes, while small subsets of genes display preferential changes in TE.

### Functional analysis of genes with differential TE upon heat stress

Next, to explore the functional implications of the genes with largely changed (adjusted *P* < 0.1) TE between the normal and heat shock conditions, we performed the gene ontology (GO) with biological processing (BP) and KEGG-pathway analysis using DAVID (Tables S4–S7).

Generally, the changes of HSP genes in RPFs are aligned well with their changes in total mRNA transcripts. Hence, genes clustered in the mostly affected TE groups include few HSP genes.

**Figure 2:**
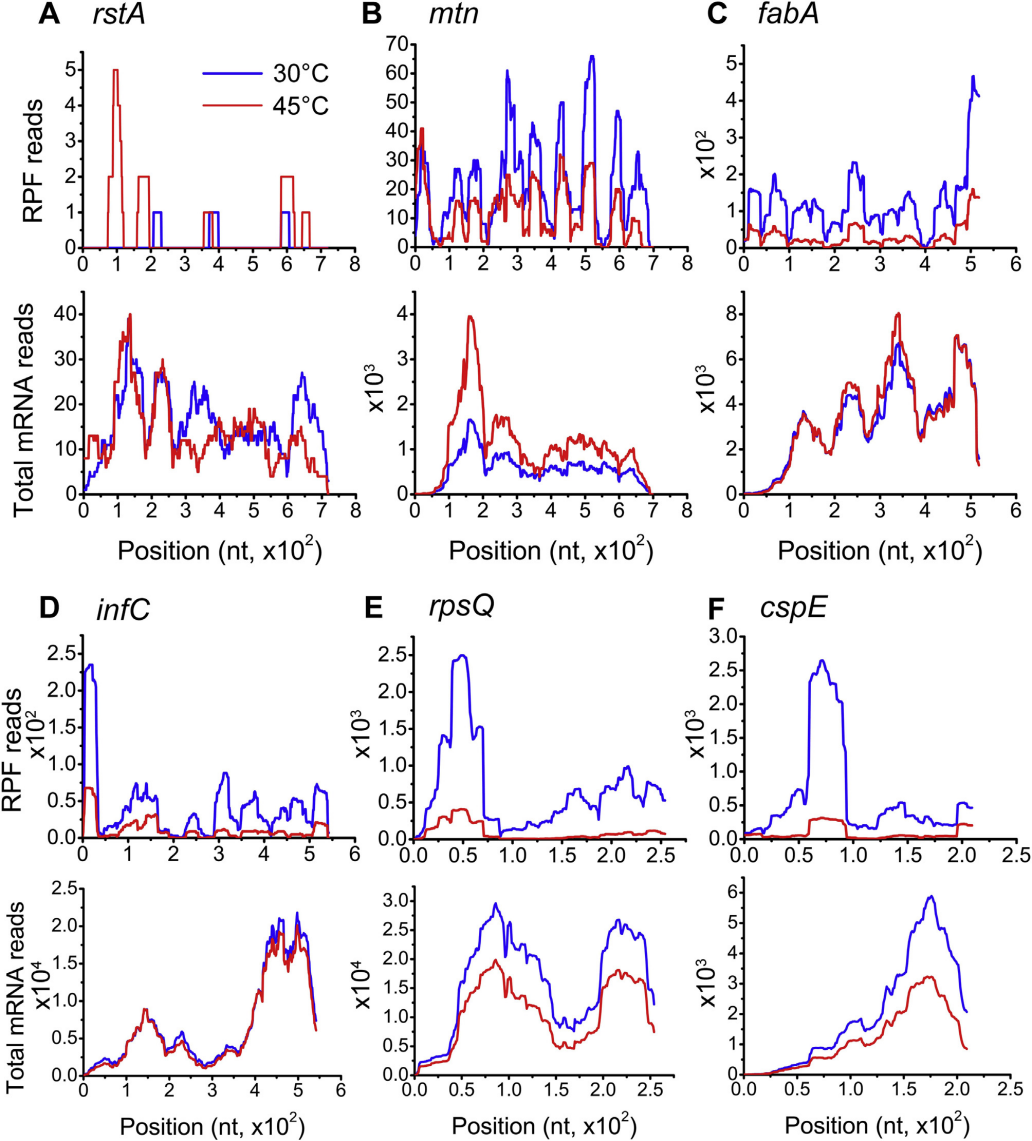
**Representative genes with large changes in translation efficiency upon heat stress** **A.** Read densities of *rstA* in RPF (upper panel) and total mRNA (bottom panel) as an example for a two-component system (with TE up-regulated upon heat stress). **B.** Read densities of *mtn*, an example for cysteine and methionine metabolism pathway (with TE up-regulated upon heat stress). **C.** Read densities of *fabA*, a representative of genes involved in fatty acid biosynthesis pathway. **D.** Read densities of *infC*, an example of translation factors. **E.** Read densities of *rpsQ*, a representative of ribosome protein genes. **F.** Read densities of *cspE*, an example of genes with down-regulation at both transcription and translation levels. On the X-axis, positions relative to the translational start site are indicated. TE, translation efficiency.

KEGG pathway analysis identified that many genes with large changes (adjusted *P* < 0.1) in TE between the normal and heat-treated conditions are involved in a few major cellular processes. For instance, heat stress showed a significant correlation with two-component system pathways (*P* = 0.019), which involve 5 genes that were identified with large increase in TE. These include *rstA* (adjusted *P* value 0.024), *frd* (adjusted *P* = 0.096), *dcuB* (adjusted *P* = 0.092), *phoB* (adjusted *P* = 0.013) and *pstS* (adjusted *P* = 8.77E−5). Bacteria often respond to environmental changes through the two-component system, which consists of a membrane-associated histidine kinase and a response regulator to regulate the expression of target genes [14]. As shown in Figure 2A, the mapped reads of RPF and total mRNA for the gene *rstA* indicate that while mRNA transcripts remain relatively constant upon heat stress, the TE (reflected by the ratio obtained with the comparison of RPF reads to mRNA reads) of *rstA* appears to be sharply increased. RstA is a regulator of the two component system, which together with the membrane-associated histidine kinase RstB responds to environmental stimulus [15]. Therefore, this observation suggests that certain stress response systems use translation regulation to control the production of effector proteins. Besides, the genes with up-regulated TE are also enriched in pathways of microbial metabolism, such as nitrogen metabolism, citrate cycle, butanoate metabolism, as well as biosynthesis of antibiotics and secondary metabolites.

**Supplementary Figure S3:**
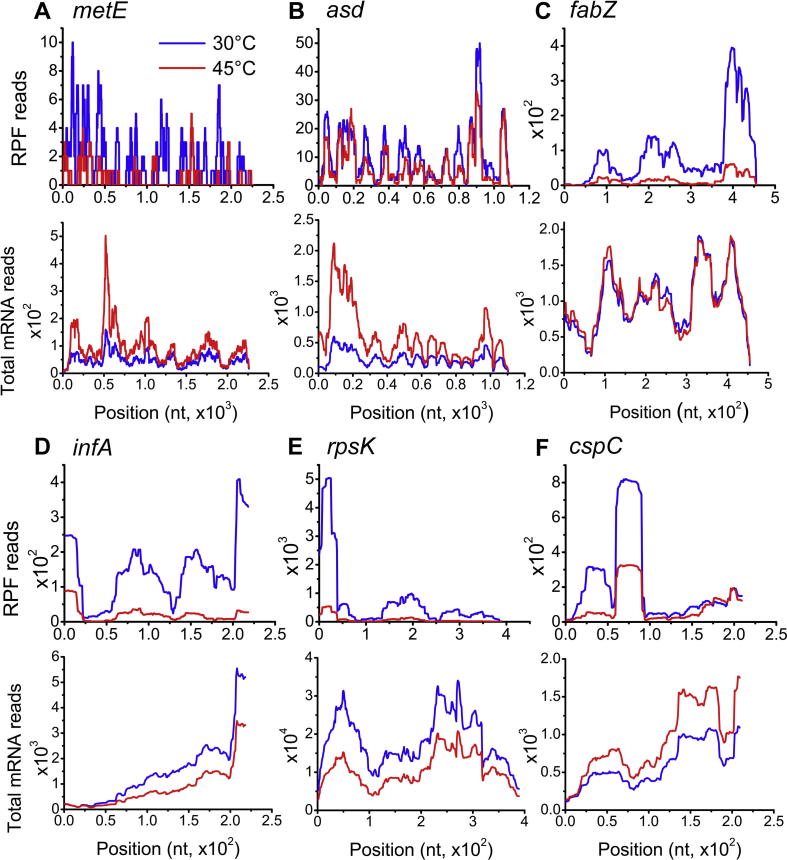
**Representative genes of *E. coli* with down-regulated translation efficiency upon heat stress**Read densities of *metE* (**A**) and *asd* (**B**) at RPF (upper panel) and total mRNA (lower panel) levels, as examples for genes involved in cysteine and methionine metabolism pathway. (**C**) Read densities of *fabZ*, an example for fatty acid biosynthesis pathway. (**D**) Read densities of *infA*, an example for genes involved in translation. (**E**) Read densities of *rpsK*, an example of genes encoding ribosomal proteins. (**F**) Read densities of *cspC*, an example for cold shock-like proteins. The start codon and stop codon locations are marked by dash lines in cyan and magenta, respectively. On the X-axis, positions relative to the translational start site are indicated.

In contrast, we found that the TE of genes related to the cell growth and amino acid biosynthesis pathways are down-regulated significantly upon heat stress. These genes include *asd* (adjusted *P* = 0.024), *metE* (adjusted *P* = 0.030), *mtn* (adjusted *P* = 0.041), *serA* (adjusted *P* = 0.037), and *ilvC* (adjusted *P* = 7.19E−4). *metE* encodes methionine synthase, while proteins encoded by *mtn* and *asd* participate in L-methionine biosynthesis via salvage pathway and de novo pathway, respectively [16], [17], [18]. The mapped reads of these genes are shown in Figure 2B (*mtn*), Figure S3A (*metE*) and Figure S3B (*asd*), respectively. Interestingly, while the transcript levels of these three genes all appear to be up-regulated, their RPFs are unexpectedly down-regulated. Given methionine is a unique amino acid required in initiation of protein synthesis, these observations suggest that down-regulation of the translation of mRNAs encoding enzymes related to biosynthesis of methionine might be part of the mechanism for the inhibition of translation initiation upon heat stress. Moreover, two genes involved in fatty acid biosynthesis (*P* = 0.17), including *fabZ* (adjusted *P* = 4.1E−6, Figure S3C) and *fabA* (adjusted *P* = 0.034, Figure 2C), also exhibit down-regulated TE.

Interestingly, four genes with down-regulated TE were found in the functional group of mRNA translation and ribosome assembly (GO analysis *P* = 0.172). These include *infC* that encodes translation initiation factor IF3 (adjusted *P* = 0.055, Figure 2D), *infA* that encodes IF1 (adjusted *P* = 0.023, Figure S3D), *rpsK* that encodes 30S ribosomal protein S11 (adjusted *P* = 0.071, Figure S3E), and *rpsQ* that encodes S17 (adjusted *P* = 0.037, Figure 2E). Notably, the transcriptional levels of *rpsQ*, *infA*, and *rpsK* are also down-regulated. The down-regulated TE of these genes upon heat stress could be reasoned that the slow-down of the production of their encoded proteins is necessary in terms of energy conservation in stress conditions, which is in agreement with previous reports [19], [20]. These observations suggest a global repression of translation upon heat stress by limiting both the transcription and translation levels of components involved in protein biosynthesis.

In addition, two genes, *tsr* (adjusted *P* = 0.037) and *fliC* (adjusted *P* = 2.43E−4) involved in taxis are among the down-regulated TE cluster. It has been reported that the decreased cell mobility could be caused by energy deficiency in stress conditions [21]. The TE down-regulation of these two genes upon heat stress, therefore, is again correlated with the cellular needs for energy conservation under stress conditions. GO analysis also revealed that a few genes in response to temperature stimulus (*P* = 0.052) showed dramatically reduced TE. These include *cspE* (adjusted *P* = 0.039; Figure 2F), *cspG* (adjusted *P* = 5.43E−6), and *cspC* (adjusted *P* = 0.043; Figure S3F), the expression of which is induced to deal with the cold stress [22].

Taken together, these data suggest the presence of multiple parallel mechanisms at translational level in bacteria to control the balance between cell growth and survival during heat shock response.

### Ribosomes accumulate in the initiation region of ORFs upon heats shock

**Figure 3:**
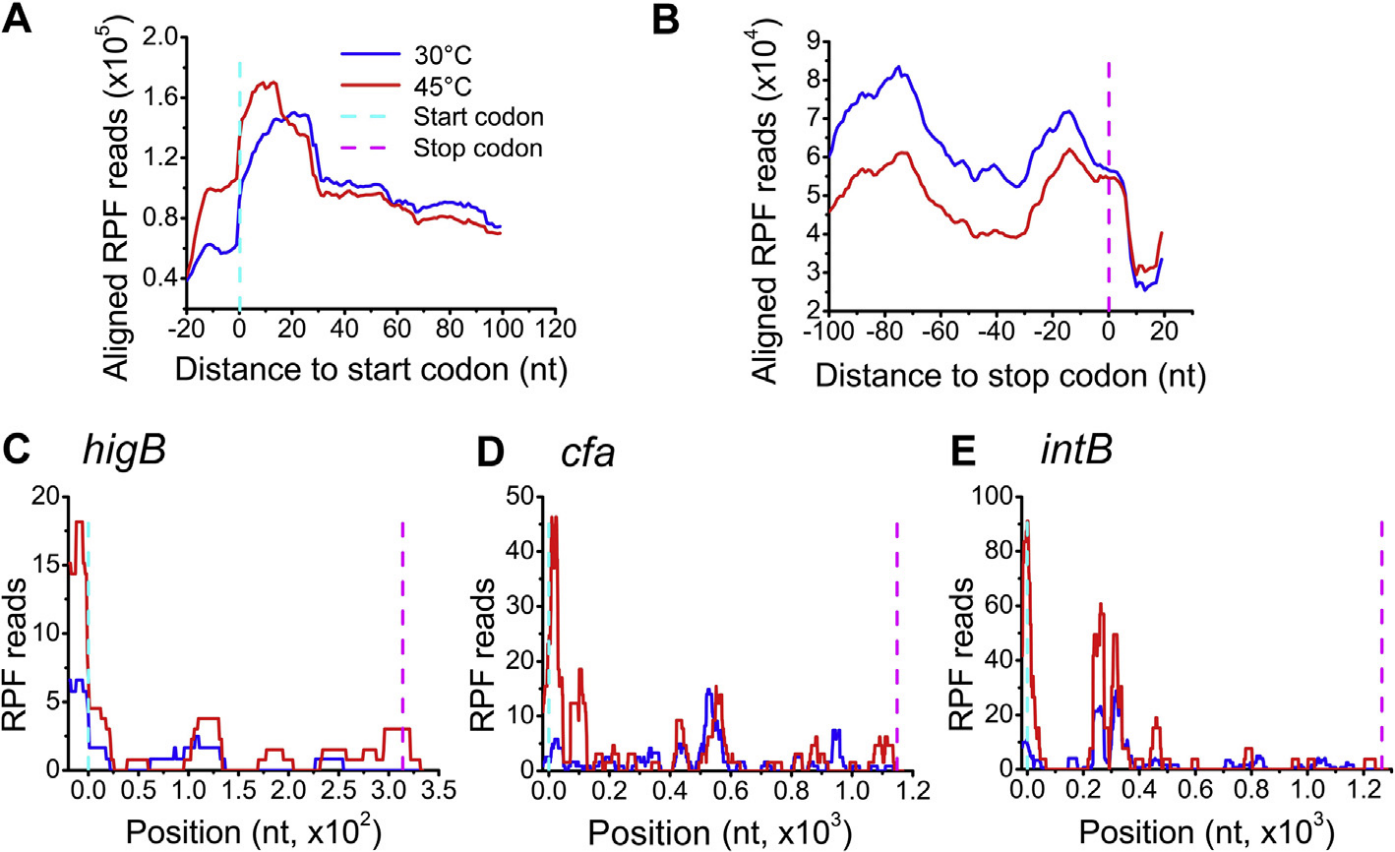
**The density of ribosome footprints around start codon increases upon heat stress** **A.** Alignments of RPF reads in the 5′UTR of all mapped genes in a 20-nucleotide distance to start codon. **B.** Alignments of the RPF reads in 3′UTR of all mapped genes in a 20-nucleotide distance to stop codon. **C.–E.** Three representative genes *higB* (C), *cfa* (D), and *intB* (E) with their ribosome footprint densities sharply increased around the start codon. The start and stop codons locations are marked by vertical dash lines in cyan and magenta respectively. On the X-axis, positions relative to the translational start site are indicated in panels C**–**E.

One advantage of ribosome profiling is its codon resolution. Therefore, we investigated whether there are any general changes in ribosome occupancy along mRNAs upon heat stress. To quantify the translation events, we calculated the average occupancy of the ribosomes around the annotated start codons and stop codons. Figure 3A and B show the averaged read distribution in the 5′UTR and the 3′UTR. Interestingly, the footprints mapped to the initiation region increased dramatically at the genome-wide level upon heat stress in *E. coli*, with the 5′-end roughly at -12 position (Figure 3A). By contrast, heat shock did not induce notable changes of ribosome occupancy in stop codon regions (Figure 3B). This observation is similar to a previous study showing that yeast ribosomes accumulate at the beginning of ORFs rapidly upon oxidative stress [23]. Heat shock resulted in about 2-fold increase in the average ribosome occupancy at the nucleotide −12 to the start codon. In addition, ribosome occupancy in the first 10 nucleotides after start codon is also about 30% higher in the heat-treated condition than the normal condition.

A few representative genes with increased ribosome occupancy in the initiation regions upon heat shock are shown in Figure 3C–E, including *higB*, *cfa* and *intB*. These data are in good agreement with previous results from eukaryotic cells [24], indicating that pausing of ribosomes at initiation and early elongation steps are a common reaction of the ribosome upon heat shock in both prokaryotes and eukaryotes. The accumulation of footprints in the initiation region of ORFs may lead to down-regulation of the global TE, proving an additional way to slow down the protein production in order to promote survival upon heat stress.

## Conclusion

Our study provides the landscape of transcription and translational regulation in response to heat shock in *E. coli*. Generally, the ribosome footprint tracks with the mRNA transcript changes, indicating that heat stress-induced gene expression reprogramming occurs primarily at transcription level. With that in mind, however, for certain small sets of genes, bacteria appear to possess specialized mechanisms, likely occurring on the ribosome, to enhance or offset the effects of transcriptional regulation. By taking advantage of the high resolution of ribosome profiling technique, we also find that ribosome footprints remarkably accumulate in the initiation regions of ORFs, as a result of impaired translation initiation or pausing of early translation elongation.

## Materials and methods

### Culture and heat exposure

100 μl of overnight culture of *E. coli* BW25113 cells was inoculated into 1 L LB media, shaking at 30 °C. When the OD_600_ of the culture reached 0.2, the culture was split into two halves, with one continuously kept at 30 °C and the other at 45 °C. When OD_600_ reached ∼0.5, 200 μg/ml chloramphenicol was added into the cultures, shaking for another 2 min before harvest.

### Lysate preparation

*Escherichia coli* lysate was prepared following the protocols described previously [10], with minor modifications. Cultures were poured onto chipped ice of the same volume and centrifuged at 4000 rpm for 10 min at 4 °C for harvest. The resulting cell pellet was washed using 10 ml resuspension buffer [10 mM MgCl_2_, 100 mM NH_4_Cl, 20 mM Tris–HCl (pH 8.0) and 1 mM chloramphenicol], and centrifuged again at 3000*g* for 5 min at 4 °C. Cell pellet of each sample was then resolved in 2 ml lysis buffer [20 mM Tris–HCl (pH8.0), 100 mM NH_4_Cl, 10 mM MgCl_2_, 0.4% Triton X-100, 0.1% NP-40, 100 U/ml of RNase-free DNase I (Roche), 0.5 U/μl of SUPERase In RNase inhibitor (Ambion) and 1 mM chloramphenicol]. Resuspended cells were poured over liquid nitrogen in 50 ml tubes, which were prepared with holes on the cap punched by a syringe needle. Then the tubes were placed at −80 °C for 30 min for nitrogen evaporation. Frozen cell pellets were pulverized using Freezer/Mill 6770 with the speed of impactor at 10 cycles per second for 2 min in two sets, transferred to a 50 ml tube, and stored at −80 °C. After nitrogen evaporation, pulverized cells in 50-ml tubes were placed in water bath at 30 °C for immediate thaw, after which the cells were kept on ice for 10 min, and then transferred into a 1.5 ml Eppendorf tube. Lysate was aliquoted into 200 μl fractions, and flash frozen by liquid nitrogen and stored at −80 °C prior to further use.

### RNA extraction and library construction

50 μl of the cell lysate per sample was used to isolate total mRNAs with Gene JETRNA purification kit (Thermo Fisher Scientific, K0731). The rRNA removal for 5 μg total mRNAs was accomplished using the Ribo-Zero™ MagneticKit for Gram-negative bacteria (Epicentre, MRZGN126). Afterward, RNAs were purified with RNA Clean & Concentrator kit (Zymo Research, R1015).

RPFs from 100 μl lysate per sample were extracted as previously described [10]. Notably, the concentration of Mg^2+^ in the lysate was supplemented to 15 mM to decrease the dissociation of 70S ribosomes. Clarified lysate was digested using 60 U MNase per one A260 unit of RNA with 5 mM CaCl_2_ in the reaction system, rotated at 1400 rpm for 1 h at 25 °C in a thermomixer. The digestion was quenched with 6 mM EGTA. RPFs were isolated with Sephacryl S400 (GE, MicroSpin S-400 HR, 27514001) and purified using Acid Phenol: Chloroform extraction method prior to storage at −80 °C for further use.cDNA libraries of total mRNA and RPFs were constructed using the ARTseq™ Ribosome Profiling Kit (Epicentre, RPHMR12126) with different index tag at the reverse primers listed in Table S8. Other primers used during the cDNA library construction were same as mentioned in the ARTseq™ Ribosome Profiling Kit (Epicentre, RPHMR12126).

### Deep sequencing

Prepared cDNA libraries were tested with Agilent 2100 bio analyzer for quality determination and then subjected to sequencing with 50-bp single end using Illumina Hiseq 2000.

### Data analysis

The 3′-adapter sequences of the reads were trimmed using the Cutadapt program [25] for both mRNA and RPF. Low-quality reads with Phred quality score less than 25 (>75% of bases) were discarded using fastx quality filter (http://hannonlab.cshl.edu/fastx_toolkit/). Reads originating from rRNAs were filtered by aligning to *E. coli* rRNA sequences using Bowtie (version 1.1.2) with no mismatch allowed. The remaining reads were then mapped to the *E. coli* genome (MG1655) using Bowtie with default parameters except “- -best –strata”. mRNA and RPF reads for each gene were counted using HTSeq-count (http://www-huber.embl.de/users/anders/HTSeq/doc/count.html). For RPFs, the reads ranging between 25 and 40 nt and uniquely mapped were deemed to be high quality and were used for further analysis.

Xtail software (https://github.com/xryanglab/xtail/releases) was used to identify genes with different TE in pairwise comparisons. In order to reduce the false positive rate, two parallel pipelines were implemented in Xtail to compare the log_2_ fold change (log_2_FC) of RPF and total mRNA (V1) or the log_2_ ratios (log_2_R) of RPF to total mRNA (V2) between two conditions. From these two parallel pipelines, the more conservative set of results (larger *P* value) were derived as the final result (final). Using this strategy, Xtail assesses discoordination between the change in mRNA abundance and total mRNA levels, theoretically equally evaluating the discoordination of a gene’s translational rate in any two conditions to be compared [13]. The final *P* values were adjusted using Benjamini–Hochberg multiple testing. Differentially-translated genes were defined here as those with an adjusted *P* < 0.1 [or false discovery rate (FDR) <0.1]. All other analyses were performed by custom python scripts. Enrichment of genes with up- or down-regulation in TE between the normal and heat-shock conditions were performed using DAVID Functional Annotation tools [26], [27].

The raw sequence data reported in this paper have been deposited in the Genome Sequence Archive [28] in BIG Data Center [29], Beijing Institute of Genomics (BIG), Chinese Academy of Sciences, under accession number CRA 000347, that is publicly accessible at http://bigd.big.ac.cn/gsa.

## Authors’ contributions

NG and XY conceived the idea and planned the project. YZ, QZ, and JF performed experiments to prepare cDNA libraries. ZX and YZ participated in bioinformatics analysis. YZ and NG drafted the manuscript. QW helped with manuscript preparation. All authors read and approved the final manuscript.

## Competing interests

The authors have declared that no competing interests exist.

## Supplementary material

Supplementary Table S1 **Statistics of sequencing reads**

Supplementary Table S2 **Quantification of the fold change at the mRNA and RPF levels for 2939 genes with more than 5 reads mapped**

Supplementary Table S3 **Genes with differential translational regulation upon heat shock (adjusted *P* < 0.1)**

Supplementary Table S4 **Gene enrichment result of up-regulated TE with GO-BP analysis**

Supplementary Table S5 **Gene enrichment result of down-regulated TE with GO-BP analysis**

Supplementary Table S6 **Gene enrichment result of up-regulated TE with KEGG pathway analysis**

Supplementary Table S7 **Gene enrichment result of down-regulated TE with KEGG pathway analysis**

Supplementary Table S8 **Primers used in library preparation at the 5’-end with index tag**
