# Supplementary material for: Ribosome Profiling Reveals Genome-wide Cellular Translational Regulation upon Heat Stress in Escherichia coli
Source: Genomics Proteomics Bioinformatics. 2017 Oct 12;15(5):324–30. doi: 10.1016/j.gpb.2017.04.005 (PMC5673677; doi:10.1016/j.gpb.2017.04.005)
Supplement: Supplementary Table S1 — Statistics of sequencing reads [file mmc1.docx]

**Table S1** **Statistics of sequencing reads**

| **Sample** | **RPF** | |  | | **mRNA** | |
| --- | --- | --- | --- | --- | --- | --- |
|  | **30°C** | **45°C** |  | | **30°C** | **45°C** |
| No. of total reads | 41,400,860 | 43,084,845 |  | 36,308,125 | | 43,500,137 |
| No. of reads after trimming | 35,474,673 | 37,044,042 |  | 35,194,410 | | 43,068,109 |
| No. of low-quality reads | 996,684 | 960,720 |  | 506,982 | | 629,488 |
| No. of reads mapped to rRNAs | 31,570,044 | 34,012,008 |  | 30,047 | | 59,699 |
| No. of reads mapped to transcriptome | 2,401,035 | 1,663,447 |  | 32,917,372 | | 39,493,934 |
| No of genes with reads mapped | 3688 | 3782 |  | 4276 | | 4285 |

*Note*: Low quality reads refer to reads with > 75% of bases having quality score < 25. RPF, ribosome protected fragment.
