# Supplementary material for: Ribosome Profiling Reveals Genome-wide Cellular Translational Regulation upon Heat Stress in Escherichia coli
Source: Genomics Proteomics Bioinformatics. 2017 Oct 12;15(5):324–30. doi: 10.1016/j.gpb.2017.04.005 (PMC5673677; doi:10.1016/j.gpb.2017.04.005)
Supplement: Supplementary Table S4 — Gene enrichment result of up-regulated TE with GO-BP analysis [file mmc4.docx]

**Table S4**  **Gene enrichment result of up-regulated TE with GO-BP analysis**

| **GO ID** | **GO term** | ***P* value** | **Genes** |
| --- | --- | --- | --- |
| 0016052 | Carbohydrate catabolic process | 0.181 | *amyA*, *dcuB*, *mtlD*, *dkgB*, *idnR*, *maa* |
| 0009061 | Anaerobic respiration | 0.231 | *fnr*, *frdC*, *nirD*, *napB* |
| 0017004 | Cytochrome complex assembly | 0.311 | *sdhC*, *napB* |
| 0006793 | Phosphorus metabolic process | 0.328 | *phoB*, *pstS* |
| 0006805 | Xenobiotic metabolic process | 0.352 | *zntR*, *cpxP* |
