# Supplementary material for: Ribosome Profiling Reveals Genome-wide Cellular Translational Regulation upon Heat Stress in Escherichia coli
Source: Genomics Proteomics Bioinformatics. 2017 Oct 12;15(5):324–30. doi: 10.1016/j.gpb.2017.04.005 (PMC5673677; doi:10.1016/j.gpb.2017.04.005)
Supplement: Supplementary Table S5 — Gene enrichment result of down-regulated TE with GO-BP analysis [file mmc5.docx]

**Table S5 Gene enrichment result of down-regulated TE with GO-BP analysis**

| **GO ID** | **GO term** | ***P* value** | **Genes** |
| --- | --- | --- | --- |
| 0009451 | RNA modification | 0.049 | *miaA*, *deaD*, *rluB*, *prmA* |
| 0009266 | Response to temperature stimulus | 0.053 | *cspE*, *cspG*, *cspC* |
| 0006826 | Iron ion transport | 0.067 | *entF*, *fecA*, *exbB* |
| 0015937 | Coenzyme A biosynthetic process | 0.119 | *entF*, *panB* |
| 0006412 | Translation | 0.173 | *infC*, *rpsQ*, *infA*, *rpsK* |
| 0009086 | Methionine biosynthetic process | 0.195 | *asd*, *metE* |
| 0006006 | Glucose metabolic process | 0.209 | *gcd*, *gntK* |
| 0015949 | Nucleobase-containing small molecule interconversion | 0.296 | *pyrG*, *mtn*, *purA* |
| 0042330 | Taxis | 0.612 | *tsr*, *fliC* |
| 0016052 | Carbohydrate catabolic process | 0.956 | *gcd*, *focA*, *rbsA* |
