# Supplementary material for: Ribosome Profiling Reveals Genome-wide Cellular Translational Regulation upon Heat Stress in Escherichia coli
Source: Genomics Proteomics Bioinformatics. 2017 Oct 12;15(5):324–30. doi: 10.1016/j.gpb.2017.04.005 (PMC5673677; doi:10.1016/j.gpb.2017.04.005)
Supplement: Supplementary Table S6 — Gene enrichment result of up-regulated TE with KEGG pathway analysis [file mmc6.docx]

**Table** **S6 Gene enrichment result of up-regulated TE with KEGG pathway analysis**

| **Pathway ID** | **Pathway name** | ***P* value** | **Genes** |
| --- | --- | --- | --- |
| eco02020 | Two-component system | 0.019 | *rstA*, *frdC*, *dcuB*, *phoB*, *pstS* |
| eco00910 | Nitrogen metabolism | 0.181 | *nirD*, *napB* |
| eco00020 | Citrate cycle (TCA cycle) | 0.181 | *frdC*, *sdhC* |
| eco00650 | Butanoate metabolism | 0.237 | *frdC*, *sdhC* |
| eco01120 | Microbial metabolism in diverse environments | 0.270 | *frdC*, *sdhC*, *nirD*, *napB* |
| eco00190 | Oxidative phosphorylation | 0.272 | *frdC*, *sdhC* |
| eco01130 | Biosynthesis of antibiotics | 0.449 | *purF*, *frdC*, *sdhC* |
| eco01200 | Carbon metabolism | 0.575 | *frdC*, *sdhC* |
| eco01110 | Biosynthesis of secondary metabolites | 0.686 | *purF*, *frdC*, *sdhC* |
| eco02010 | ABC transporters | 0.747 | *dppA*, *pstS* |
| eco01100 | Metabolic pathways | 0.855 | *amyA*, *purF*, *frdC*, *sdhC*, *waaR* |
