# Supplementary material for: Ribosome Profiling Reveals Genome-wide Cellular Translational Regulation upon Heat Stress in Escherichia coli
Source: Genomics Proteomics Bioinformatics. 2017 Oct 12;15(5):324–30. doi: 10.1016/j.gpb.2017.04.005 (PMC5673677; doi:10.1016/j.gpb.2017.04.005)
Supplement: Supplementary Table S7 — Gene enrichment result of down-regulated TE with KEGG pathway analysis [file mmc7.docx]

**Table S7 Gene enrichment result of down-regulated TE with KEGG pathway analysis**

| **Pathway ID** | **Pathway name** | ***P* value** | **Genes** |
| --- | --- | --- | --- |
| eco00270 | Cysteine and methionine metabolism | 0.065 | *asd*, *metE*, *mtn* |
| eco00030 | Pentose phosphate pathway | 0.069 | *gcd*, *edd*, *gntK* |
| eco01100 | Metabolic pathways | 0.080 | *fabZ*, *miaA*, *asd*, *ilvC*, *fabA*, *purA*, *pyrG*, *gcd*, *panB*, *metE*, *mtn*, *edd*, *gntK*, *serA*, *epd* |
| eco01230 | Biosynthesis of amino acids | 0.087 | *asd*, *metE*, *mtn*, *serA*, *ilvC* |
| eco00061 | Fatty acid biosynthesis | 0.175 | *fabZ*, *fabA* |
| eco01110 | Biosynthesis of secondary metabolites | 0.248 | *miaA*, *gcd*, *panB*, *asd*, *metE*, *gntK*, *ilvC* |
| eco01212 | Fatty acid metabolism | 0.267 | *fabZ*, *fabA* |
| eco00770 | Pantothenate and CoA biosynthesis | 0.278 | *panB*, *ilvC* |
| eco01130 | Biosynthesis of antibiotics | 0.316 | *gcd*, *asd*, *gntK*, *serA*, *ilvC* |
| eco01210 | 2-Oxocarboxylic acid metabolism | 0.320 | *asd*, *ilvC* |
| eco00260 | Glycine, serine and threonine metabolism | 0.415 | *asd*, *serA* |
| eco01200 | Carbon metabolism | 0.476 | *edd*, *gntK*, *serA* |
| eco03010 | Ribosome | 0.692 | *rpsQ*, *rpsK* |
| eco01120 | Microbial metabolism in diverse environments | 0.702 | *asd*, *edd*, *gntK*, *serA* |
| eco02010 | ABC transporters | 0.729 | *znuA*, *potA*, *rbsA* |
| eco02020 | Two-component system | 0.895 | *tsr*, *fliC* |
