# Supplementary material for: Ribosome Profiling Reveals Genome-wide Cellular Translational Regulation upon Heat Stress in Escherichia coli
Source: Genomics Proteomics Bioinformatics. 2017 Oct 12;15(5):324–30. doi: 10.1016/j.gpb.2017.04.005 (PMC5673677; doi:10.1016/j.gpb.2017.04.005)
Supplement: Supplementary Table S8 — Primers used in library preparation at the 5’-end with index tag [file mmc8.docx]

**Table S8 Primers used in library preparation with index tag**

| **Sample** | **Reverse primer** |
| --- | --- |
| RPF_45°C | 5′CAAGCAGAAGACGGCATACGAGATCGTGATGTGACTGGAGTTCAGACGTGTGCTCTTCCGATCT 3′ |
| RPF_30°C | 5′CAAGCAGAAGACGGCATACGAGATGCCTAAGTGACTGGAGTTCAGACGTGTGCTCTTCCGATCT 3′ |
| mRNA_45°C | 5′CAAGCAGAAGACGGCATACGAGATCACTGTGTGACTGGAGTTCAGACGTGTGCTCTTCCGATCT 3′ |
| mRNA_30°C | 5′CAAGCAGAAGACGGCATACGAGATGATCTGGTGACTGGAGTTCAGACGTGTGCTCTTCCGATCT 3′ |

*Note*: The forward primer is 5′AATGATACGGCGACCACCGAGATCTACAC3′. RPF, ribosome protected fragment.
